# Association of engagement in cultural activities with cause-specific mortality determined through an eight-year follow up: The HUNT Study, Norway

**DOI:** 10.1371/journal.pone.0248332

**Published:** 2021-03-11

**Authors:** Bente I. Løkken, Dafna Merom, Erik R. Sund, Steinar Krokstad, Vegar Rangul

**Affiliations:** 1 Nord University, Levanger, Norway; 2 School of Health Science, Western Sydney University, Sydney, New South Wales, Australia; 3 HUNT Research Centre, Department of Public Health and Nursing, Faculty of Medicine and Health Sciences, Norwegian University of Science and Technology (NTNU), Levanger, Norway; 4 Levanger Hospital, Nord-Trøndelags Hospital Trust, Levanger, Norway; 5 Norwegian Resource Centre for Arts and Health, Levanger, Norway; Sciensano, BELGIUM

## Abstract

Participation in cultural activities may protect against cause-specific mortality; however, there is limited knowledge regarding this association. The present study examines the association between participation in a range of receptive and creative cultural activities and risk of cardiovascular disease- and cancer-related mortality. We also examined whether participation in such activities and influence by gender have on this association. We followed 35,902 participants of the Nord-Trøndelag Health Study (HUNT3) of Cardiovascular-Disease and Cancer Mortality from 2006–08 to 2016. Cox proportional-hazards regression was used to estimate the risk of specific mortality based on baseline cultural participation. During the eight-year follow-up, there were 563 cardiovascular-disease- and 752 cancer-related deaths among the sample (292,416 person years). Risk of cardiovascular-disease mortality was higher among non-participants in associations/club meetings (22%) and outdoor activities (23%), respectively, as well as non-attendees of art exhibitions (28%). People who engaged in music, singing, and theatre had a 27% reduced risk of cancer-related mortality when compared to non-participants. Among women, participating in associations/club meetings reduced the risk of cardiovascular-disease mortality by 36%. Men who participated in music, singing, and theatre had a 33% reduced risk of cancer mortality. Overall, a reduced risk of cardiovascular-disease mortality was associated with engaging in creative activities on weekly basis to less than twice per week. For both genders, participating in creative activities less than once a week reduced cardiovascular-disease mortality risk by 40% and 33%, respectively. For the overall sample, participating > 2 times per week in combined receptive and creative activities reduced cancer-related mortality by 29%. Participating frequently in both receptive and creative activities cultural activities was associated with lower risks of CVD and cancer-related mortality. Our data suggest that, to counteract the public health burden of cardiovascular disease- and cancer mortality, policies and initiatives to increase citizens’ participation in cultural activities should be considered.

## Introduction

Non-communicable diseases (NCDs) contribute to approximately 40.5 million (71%) of all deaths globally [1], and to almost half of the disease burden in low- and middle- income countries [2]. Cardiovascular diseases (CVDs) and cancer are the leading causes of NCD-related deaths, accounting for 44% and 22%, respectively [1]. CVDs and cancer are complex and multi-causal, but lifestyle plays an important role in the prevention and management of these diseases. Consequently, preventive efforts have mainly encouraged smoking cessation, avoiding excessive alcohol intake, healthy eating, and leading a physically active lifestyle [1, 3].

Interest in the association between participation in cultural activities and health outcomes has increased in recent years. Cultural activities include everyday events performed for enjoyment, entertainment, recreation, or to contribute to society [4]. Such activities can provide opportunities for social and physical engagement [5] and, hence, may impact the population burden of major chronic diseases such as CVDs and cancer. A lack of social relationships is strong predictor of premature mortality [6–8], is detrimental to cancer survival [6, 9], and can increase the risk of coronary heart disease and stroke by a degree similar to that found for other classic lifestyle risk factors [10]. Cultural profiles and consumption patterns vary significantly across social contexts [11]. Participation in cultural activities can be ‘passive’ or ‘active’ (i.e. ‘receptive’ or ‘creative’, respectively); passive participation includes being an attendee or spectator, while active participation includes actively engaging in creative activities by doing or performing. Creative cultural participation seems to be more common than receptive attendance [12]. However, there has been a lack of research regarding the effect cultural activities can have on population health and longevity.

Cultural activities can have health-enhancing therapeutic effects; however, most related studies have involved small sample sizes and were conducted in clinical contexts [13–15] (including research on patients with CVDs [16] and cancer [17–19]). This has limited the generalisability of the findings to the public health context [13, 20, 21]. Existing longitudinal evidence is characterised by fragmented approaches and a focus on the health benefits of specific activities; for example, dancing seems to reduce the risk of CVD-related mortality [22], and attending cultural events to reduce the risk of cancer-related mortality [23]. Epidemiological studies in this field have rarely examined a person’s cultural lifestyle as a whole in relation to investigating cause-specific mortality in the same sample [12, 14] and, to our knowledge, the association between receptive and creative cultural activities and cause-specific mortality has not been previously examined. Thus, evidence from population-based samples concerning the effects of participation in a wide-range of receptive and creative activities is important for establishing the public health significance of such activities. A Scandinavian study conducted by Väänänen et al. in 2009 explored the association between cultural engagement (arts and culture, activities in associations, societal action, reading literature, and studying) and all cause- and cause specific mortality, among full-time employees [24]. Intermediate and high engagements in such activities reduced the risk of CVD-related mortality but not cancer-related mortality. We are following up exploring this hypothesis in a total adult population cohort that is not limited to the workforce, with participation in cultural life measured by a range of receptive and creative activities in a Norwegian setting.

Data from the Nord-Trøndelag Health Study (HUNT) can be linked to the Norwegian Cause of Death Registry and affords the profiling of individuals’ cultural patterns in terms of the risk of CVD- and cancer-related mortality. Using these data, the present study aimed to: 1) identify the types of cultural activities that protect against CVD- and cancer-related mortality; 2) assess whether the number of receptive and creative activities a person engages in, including weekly frequency of participation, are associated with CVD- and cancer-related mortality; and 3) explore possible gender differences between these three quantifiers (type, number, and weekly frequency).

## Materials and methods

### Study population

HUNT is a longitudinal population health study that comprised four cross-sectional surveys. Participation in the surveys was voluntary. The present study uses data from the third HUNT survey (HUNT3, 2006–2008), in which all residents of the north part of Trøndelag County (n = 93,860) who were aged ≥ 20 years were invited to participate; in total, 50,807 (response rate = 54.1%) participated [25]. Participants were given a self-report questionnaire (Q1; mailed with the invitation to participate) and were invited to a clinical examination. Q1 included questions concerning participants’ socio-demographic characteristics, health behaviours and diseases (both physical and mental), and social relationships. At the clinical examination, a second questionnaire (Q2), which concerned cultural activities [26], was distributed with a pre-paid envelope; this was to be completed at home and returned by mail. Overall, 41,198 participants (response rate = 81%) returned Q2; of these, 2.4% (984) did not answer any of the questions concerning receptive or creative activities and were excluded, meaning our baseline sample comprised 40,214 participants.

The participants signed written consent forms, which included approval to link their information to national registers [27]. This study was approved by the Regional Committees for Medical Research and Health Research Ethics in Norway (ref. no.: 2016/282/REK midt).

### Cultural participation

Cultural participation was assessed using two validated questions concerning receptive and creative activities, respectively. These questions were proven to be sufficient for public health research [28].

The receptive activity question was ‘How often in the last six months have you attended: 1) a museum/art exhibition; 2) a concert, theatre, or film; 3) a church/chapel; 4) a sports event?’ The response options were: ‘more than three times a month’, ‘1–3 times a month’, ‘1–6 times in the last six months’, and ‘never’. We dichotomised the responses into ‘never’ and ‘ever’. Then, for each participant, we summarised all of activities he/she reported attending, which reflected the diversity of their engagement. Summing the scores across all receptive activities produced a range from 4 (attending all receptive activities) to 0 (answering ‘never’ to all). Few participants reported attending more than three activities; thus, we created the category ‘3–4 activities’. Next, a score representing weekly frequency of participation in the receptive activities was assigned by giving each response option a weekly score: ‘more than three times a month‘ received a score of 1 (i.e. approximately once a week), ‘1–3 times a month’ was scored 0.5, ‘1–6 times in the last six months’ was scored 0.25, and ‘never’ was scored zero. After summing the scores across all receptive activities, we used quartiles to reflect weekly engagement: the lowest quartile represented ‘never to seldom’ (score: 0–0.25), the second quartile represented ‘every other week or less than once per week’ (0.5–0.99), the third quartile represented ‘once to less than twice per week’ (1–1.99), and the highest quartile represented ‘2–4 times per week’ (2–4).

Engagement in creative activities was measured using the question: ‘How often in the last six months have you participated in: 1) an association or club meeting/activity, 2) music, singing, or theatre, 3) parish work, 4) outdoor activities, 5) dancing, 6) sports or exercise?’ For this research, participation in ‘sports or exercise’ was excluded because exercise is a subtype of physical activity (PA), which was assessed as a covariate in Q1. The response options were: ‘more than once a week’, ‘once a week’, ‘1–3 times a month’, ‘1–5 times in the last six months’, and ‘never.’ As above, we dichotomised these categories into ‘never’ and ‘ever’. Then, for assessing diversity of participation in creative activities we summarised, for each participant, the number of activities he/she reported engaging in, ranging from 5 (participation in all creative activities) to 0 (never). We created three categories overall by merging those who performed 3–5 activities into a single category. Next, an index reflecting weekly participation was created by giving each response option a score: ‘more than once a week’ and ‘once a week’ received a score of 1; ‘1–3 times a month’ was scored 0.5, ‘1–5 times in the last six months’ was scored 0.25, and ‘never’ was scored zero. The sum of the scores across all creative activities was divided into quartiles: the lowest quartile represented ‘never to seldom’ (0–0.25), the second quartile represented ‘every other week or less than once peer week’ (0.5–0.99), the third quartile represented ‘once to less than twice per week’ (1–1.99), and the highest quartile represented ‘2–5 times per week’ (2–5).

Finally, we examined the total number of activities each participant engaged in during the past six months, combining all types of receptive and creative activities. The highest score was 9 (participation in all four receptive and five creative activities), and the lowest was 0 (never). We created seven categories by merging those who performed 7–9 activities into a single category. Similarly, combined weekly participation was created by summing weekly participation in each activity and dividing it into quartiles: the lowest quartile represented ‘never to seldom’ (0–0.25), the second quartile represented ‘every other week to less than once per week’ (0.5–0.99), the third quartile represented ‘once to less than twice per week’ (1–1.99), and the highest quartile represented ‘2–9 times per week’ (2–9).

Participants who did not respond to any of the receptive and creative activity questions were considered to have provided missing data for the cultural participation module (n = 984), and were excluded. Participants who provided only one response across the receptive and creative activity questions were not considered to have provided missing data, under the assumption that they only provided answers if they participated in the specific activity, given it was a self-completed questionnaire. This resulted in 1,228 and 1,347 participants being recorded as never participating in any receptive activity and any creative activity, respectively. As a result, our descriptive analysis of the sample included 40,214 participants.

### Mortality

The study data were linked to the Norwegian Cause of Death Registry. These mortality data are based on death certificates reported by doctors, who are required to report the cause of death in accordance with the International Classification of Diseases (ICD-10). Both the degree of coverage and completeness are high, with medical information available for over 98% of all deaths [29]. For the present research, the cause-specific outcomes were CVD- and cancer-related deaths, for which the ICD-10 codes are ‘I00-99’ and ‘C00-97’, respectively.

### Covariates

The following socio-demographic characteristics were considered confounders: age, gender, marital status, and socioeconomic status (SES) determined based on occupation). Age was categorised into 10-year categories, beginning at 20–29 years and ending at 80+ years. Ten occupation types, listed based on the ISCO88 classification [30], were collapsed into three categories: low (‘elementary occupations’), medium (‘clerks’, ‘service workers and ship and market sales workers’, ‘skilled agriculture and fishery workers’, ‘craft and related trades workers’ and ‘plant and machine operators and assemblers’), and high level (‘legislators, senior officials, and managers’, ‘professionals’, ‘technicians and associate professionals’, and ‘armed forces and unspecified’). Of the participants who provided missing information regarding occupation, 1,442 (4.0%) were categorised as having elementary occupations because the data showed they were young (possibly students and not working) or old (likely retired). Marital status, which featured nine response options, was dichotomised into ‘being in a relationship’ (married, registered partner) or ‘other’ (unmarried, widow(er), divorced, separated, separated partner, divorced partner, and surviving partner). Health-related confounders comprised longstanding illness and a range of health behaviours. Q1 included one question concerning having a longstanding illness: ‘do you suffer from longstanding (at least one year) illness or injury of a physical or psychological nature that impairs your functioning in daily life?’ (response: ‘yes’ or ‘no’). Smoking status was reported as ‘never’, ‘former’, or ‘daily’. Alcohol consumption (number of units of beer, wine, and spirits consumed in the seven days preceding the survey) was calculated and categorised into ‘never’ (0 units/week), low (1–6 units/week), and high (≥ 7 units/week). For PA, we calculated metabolic equivalents (METs), which reflected activity level in min per week, based on frequency, duration, and intensity. This was divided into two levels: above and below the international recommendation of at least 150 min/week of moderate-to-vigorous intensity, respectively [31]; this corresponds to 500 MET minutes per week. A continuous body mass index (BMI) variable was constructed based on the height and weight variables measured in the clinical examination; participants were categorised into three groups: ‘normal weight’ (< 18–24.9), ‘overweight’ (25.0–29.9), or ‘obese’ (≥ 30).

### Statistical analysis

First, we cross-tabulated our primary exposures (cultural activity) with likely confounding factors for each gender. The relationships between cultural participation and cause-specific mortality were analysed using multivariable time to event models. The Cox proportional hazard regression model was applied, and hazard ratios (HRs) and 99% confidence intervals (CIs) were reported. These HRs represented the ratios between various groups regarding the probability of dying from CVD and cancer, respectively. Proportional hazard assumptions were tested. Based on the large number of hypothesis tests performed, 99% CIs were used to reduce the probability for type-1 error rate. We developed estimates for each receptive and creative activity. We also examined the effect of diversity of participation (based on the number of different activities participants engaged in) and level of participation (measured using weekly frequency). These explanatory quantifiers were explored within each mode of cultural activity (i.e. receptive and creative), and also for all cultural activities combined. Models with missing category on the covariates were also specified, and finally, we fitted models in which we removed participants who died within the first two years (to circumvent problems regarding reverse causation). Hence the association between cultural participation and cause-specific mortality included comprised 35,902 individuals, as 9.9% (3,996) provided missing data for any covariates and 0.87% (316) died from all-cause mortality.

Causal directed acyclic graphs were used to guide the modelling strategy. First, models were run with each cultural activity only, adjusting for age and gender. Second, SES and marital status were added to the model, and the third model included longstanding illness. In the final model, lifestyle covariates were added (alcohol consumption, smoking status, PA, BMI). In addition, gender-specific analyses were performed for all of these models. Person-time was determined for each participant based on the period from the date of baseline participation to date of death, of loss to follow up, or December 31^st^, 2015, whichever came first. IBM SPSS version 24 (SPSS, inc., Chicago, Illinois) was used to perform the analyses.

## Results

### Descriptive analyses

Overall, data for 17,606 (43.8%) men and 22,608 (56.2%) women (mean age: 55 and 53 years, respectively) were included in the descriptive analyses. Table 1 shows the gender-specific distribution of the participants in terms of the covariates (first column), and among those who performed each receptive cultural activity. Strong gender differences were observed for occupational class, marital status, alcohol use, and BMI. Regarding activities, notable gender differences were observed for attending places of worship (church/chapel) and concert/theatre/film. Women with low-level occupations tended to participate in these activities more than men with low-level occupations; similarly, married women tended to visit places of worship more than married men (67% vs. 28%). Never using alcohol and having normal BMI were more prevalent among women than men across all receptive activities, whereas gender differences regarding smoking status and longstanding illness were small across each receptive activity.

**Table 1:** Gender-specific distribution (%) of the participants in terms of the covariates and participation in each receptive cultural activity (n = 40,214). The HUNT Study (2006–08).

		*All*		Museum/art exhibition		Concert, theatre, film		Church/chapel		Sports event	
		Men	Women	Men	Women	Men	Women	Men	Women	Men	Women
**Total**		17,606 (43.8)	22,608 (56.2)	4,913 (27.9)	7,210 (31.9)	10,008 (56.8)	14,591 (64.5)	9,581 (54.4)	12,957 (57.3)	9,182 (52.2)	9,165 (40.5)
**Mean age ± std**.		55.2 ± 15.0	53.5 ± 16.1	54.8 ± 14.1	52.2 ± 14.6	51.6 ± 14.8	49.5 ± 15.1	56.5 ± 14.4	54.4 ± 15.9	51.9 ± 14.3	46.9 ± 14.1
**Occupation level**	Low	4.7	15.5	4.7	7.9	4.7	10.5	3.9	14.0	4.7	9.5
	Medium	60.0	51.7	40.3	40.2	50.8	47.5	58.6	51.4	54.4	48.2
	High	35.4	32.8	55.0	51.9	44.5	42.0	37.5	34.6	40.8	42.3
**Marital status**	Marriage^†^	64.8	57.6	69.0	61.7	64.6	58.5	29.4	61.8	65.0	59.8
	Other	35.2	42.4	31.0	38.3	35.4	41.5	70.6	48.2	35.0	40.2
**LLI**	Yes	41.4	41.9	35.3	36.4	34.1	35.5	41.0	41.5	34.7	31.4
	No	58.6	58.1	64.7	63.6	65.9	64.5	59.0	58.5	65.3	68.6
**Alcohol, units/week**	Never	15.1	27.9	11.3	18.6	10.4	20.7	16.4	29.6	10.5	20.2
	0.5–6.5	74.9	69.2	75.9	77.2	78.1	76.0	75.6	68.0	79.3	77.1
	≥ 7	10.0	2.9	12.8	4.2	11.5	3.3	8.1	2.5	10.2	2.7
**Cigarette smoking**	Never	40.1	45.2	45.1	48.7	45.9	47.0	43.2	49.9	46.6	48.2
	Former	38.1	30.4	38.0	32.7	34.6	31.1	38.5	29.6	33.8	28.7
	Daily	21.8	24.4	16.9	18.7	19.5	21.9	18.3	20.5	19.5	23.1
**Physical activity, MET**	< 2.5	60.2	56.4	53.9	49.8	55.9	51.2	59.0	56.4	52.9	47.6
	≥ 2.5	39.8	43.6	46.1	50.2	44.1	48.8	41.0	43.6	47.1	52.4
**BMI**	Normal	24.6	38.4	25.4	42.5	25.4	41.4	23.8	36.6	25.1	43.6
	Overweight	53.1	38.1	53.5	37.5	53.9	37.6	54.1	39.5	53.9	36.6
	Obesity	22.4	23.5	21.0	20.0	20.6	21.1	22.2	23.9	21.0	19.8

^†^Marriage/relationship

MET: metabolic equivalent; LLI: Limiting longstanding illness.

Table 2 shows gender-specific distribution of participation in each creative activity in terms of each covariate. For each creative activity, there was a higher representation of women with low-level occupations than men with low-level occupations. Strong gender differences regarding marital status were observed for participation in ‘parish work’ and ‘dance’, with more married men than married women engaging in these activities. Gender differences regarding alcohol, smoking, and BMI were noted across all activities, with more women than men never drinking alcohol, never smoking, and being of normal weight.

**Table 2:** Gender-specific distribution (%) of participants in terms of engagement in each creative cultural activity (n = 40,214). The HUNT Study (2006–08).

		Association or club meeting/activity		Music, singing, theatre		Parish work		Outdoor activities		Dance	
		Men	Women	Men	Women	Men	Women	Men	Women	Men	Women
**Total**		7,136 (40.5)	9,373 (41.5)	3,364 (19.1)	4,404 (19.5)	802 (4.6)	1,410 (6.2)	14,426 (81.9)	16,999 (75.2)	5,683 (32.3)	8,443 (37.3)
**Mean age ± std**.		53.9 ± 13.8	54.5 ± 15.3	54.4 ± 15.2	49.8 ± 16.0	56.6 ± 14.8	57.0 ± 15.5	54.1 ± 14.4	51.1 ± 14.8	54.9 ± 13.3	50.2 ± 14.2
**Occupation level**	Low	3.7	10.9	4.7	9.7	4.5	13.7	4.5	11.4	4.3	10.7
	Medium	50.0	48.2	49.9	45.0	51.0	44.5	56.9	50.0	54.7	50.5
	High	46.3	40.9	45.3	45.3	44.5	41.8	38.7	38.6	41.0	38.9
**Marital status**	Marriage^†^	69.5	63.0	67.3	57.7	78.9	68.3	65.5	60.2	67.3	59.8
	Other	30.5	37.0	32.7	42.3	21.1	31.7	34.5	39.8	32.7	40.2
**LLI**	Yes	36.7	39.5	38.9	36.3	39.8	44.8	38.3	37.0	36.4	34.9
	No	63.3	60.5	61.1	63.7	60.2	55.2	61.7	63.0	63.6	65.1
**Alcohol, units/ week**	Never	12.7	25.8	14.2	24.2	46.3	55.4	13.0	23.1	7.4	16.7
	0.5–6.5	77.1	71.5	75.0	72.9	50.2	43.3	76.6	73.7	81.7	79.7
	≥ 7	10.2	2.7	10.8	3.0	3.5	1.3	10.4	3.2	10.9	3.6
**Cigarette smoking**	Never	46.8	50.3	42.9	50.7	54.2	66.3	42.5	45.6	43.5	44.8
	Former	34.7	9.8	36.7	28.1	30.3	22.6	36.9	30.9	36.4	30.4
	Daily	18.5	19.8	20.5	21.3	15.5	11.1	20.7	23.5	20.1	24.9
**Physical activity, MET**	< 2.5	57.8	54.9	57.6	52.0	61.7	62.2	56.7	50.5	55.8	48.1
	≥ 2.5	43.1	45.1	42.4	48.0	38.3	37.8	43.3	49.5	44.2	51.9
**BMI**	Normal	23.7	36.7	24.8	40.2	25.2	35.6	24.7	40.9	23.5	42.0
	Overweight	53.8	39.0	53.8	36.9	51.1	37.7	54.0	38.2	55.0	38.5
	Obesity	22.5	24.3	21.4	22.9	23.8	26.7	21.3	20.8	21.5	19.5

^†^Marriage/relationship

MET: metabolic equivalent; LLI: Limiting longstanding illness

### Association with cause-specific mortality

The mean duration of follow-up was 8.15 years, resulting in a total of 292,416 person years. During this time, 235 (1.04%) women and 328 (1.86%) men died from CVD-related issues, and 313 (1.38%) women and 439 (2.49%) men died from cancer-related issues (Table 3).

**Table 3:** Total and gender-specific associations (based on adjusted^†^ hazard ratios and 99% confidence intervals) between participation in one or more receptive/creative cultural activities and cardiovascular-disease- and cancer-related mortality (n = 35,902). The HUNT Study (2006–08).

			Receptive activities				Creative activities				
		Deaths/ person years	Museum/art exhibition	Concert, theatre, film	Church/ chapel	Sports event	Association or club meeting/activity	Music, singing, theatre	Parish work	Outdoor activities	Dance
**Participants**			11,305	22,870	20,232	17,082	15,143	7,167	1,970	28,910	13,083
			(31.5)	(63.7)	(56.4)	(47.6)	(42.2)	(20.0)	(5.5)	(80.5)	(36.4)
			**HR (99% CI)**				**HR (99% CI)**				
**CVD**	All	563/292416	0.72	0.85	1.00	0.83	0.78	1.07	0.91	0.77	0.82
			(0.53–0.97)	(0.66–1.08)	(0.80–1.26)	(0.63–1.09)	(0.62–0.99)	(0.81–1.43)	(0.57–1.46)	(0.61–0.98)	(0.62–1.10)
	Men	328/129851	0.74	0.75	0.97	0.75	0.90	1.04	1.40	0.74	0.79
			(0.51–1.07)	(0.54–1.03)	(0.73–1.31)	(0.55–1.04)	(0.65–1.24)	(0.72–1.50)	(0.78–2.51)	(0.54–1.01)	(0.55–1.14)
	Woman	235/162565	0.71	1.04	1.02	1.06	0.64	1.10	0.53	0.83	0.83
			(0.43–1.17)	(0.71–1.52)	(0.72–1.45)	(0.64,1.74)	(0.45–0.92)	(0.69,1.76)	(0.24–1.17)	(0.57–1.19)	(0.52–1.34)
**Cancer**	All	752/292416	0.98	0.90	0.85	0.95	0.88	0.73	0.72	0.82	0.84
			(0.78–1.22)	(0.73–1.10)	(0.70–1.03)	(0.77–1.17)	(0.72–1.08)	(0.56–0.97)	(0.45–1.16)	(0.66–1.02)	(0.68–1.05)
	Men	439/129851	0.83	0.84	0.84	0.95	0.91	0.67	0.73	0.87	0.76
			(0.61–1.13)	(0.64–1.10)	(0.66–1.08)	(0.73–1.23)	(0.70–1.20)	(0.47–0.96)	(0.37–1.46)	(0.65–1.17)	(0.57–1.02)
	Woman	313/162565	1.18	0.98	0.86	0.93	0.86	0.83	0.71	0.74	0.96
			(0.84–1.65)	(0.71–1.35)	(0.64–1.16)	(0.65–1.33)	(0.64–1.17)	(0.54–1.27)	(0.37–1.37)	(0.54–1.03)	(0.69–1.35)

^†^ Adjusted for: age and gender, occupation and marital status, limiting longstanding illness, and behavioural lifestyle factors (smoking, alcohol consumption, physical activity, and body mass index; ref.: never).

CI: confidence interval; CVD: cardiovascular disease; HR: hazard ratio

### Individual activities

The relationships between each cultural activity and the respective dependent variables of CVD- and cancer-related mortality were examined individually (Table 3). The results of the fully adjusted multivariable analysis revealed that, among receptive activities, only attending museum/art exhibitions positively influenced CVD-related mortality; the fully adjusted model showed a significantly lower risk (HR: 0.72; 99% CI: 0.53–0.97) for the whole population in this regard. Gender-specific analyses revealed that neither women nor men experienced a significant effect of participating in any of the receptive activities.

Several creative activities lowered the risk of CVD-related mortality; association or club meetings/activities reduced CVD-related mortality by 22% (adjusted HR: 0.78; 99% CI: 0.62–0.99), and outdoor activities produced a reduction of 23% (adjusted HR: 0.77; 99% CI: 0.61–0.98). Gender-specific analysis revealed that the only activity that lowered the risk of CVD-related mortality in women was participating in association or club meetings/activities (risk reduction: 36%; adjusted HR: 0.64; 99% CI: 0.45–0.92). In contrast, no creative activities were found to reduce CVD-related mortality among men.

Only music, singing, and theatre was found to significantly influence cancer-related mortality (risk reduction: 27%; adjusted HR: 0.73; 99% CI: 0.56–0.97). However, gender-specific analysis showed that the protective effect of music, singing and theatre was not present for women but was strong for men, at 33% (adjusted HR: 0.67; 99% CI 0.47–0.96).

### Diversity of participation

Diversity in participation was not found to be an important determinant of CVD- or cancer-related mortality for either men or for women (S1–S6 Figs). In the fully adjusted model CVD-related mortality among those who participated in two, three or more receptive or creative activities did not significantly differ when compared to those who participated in less than two activities. A similar pattern was found in gender-specific analysis, which suggested that engaging in several different activities did not produce any extra benefits regarding CVD-related mortality. In contrast, the total number of receptive and creative activities engaged in impacted cancer-related mortality, and gender-specific analyses revealed that this influenced men’s longevity (S6 Fig). Notably, participating in increasing numbers of receptive activities did not seem to moderate a reduction in risk of CVD- or cancer-related mortality (see S3 and S4 Figs). The differences appeared between participants who participated in one activity and those who did not participate at all.

### Weekly frequency of participation

Table 4 shows the results of the fully adjusted models presenting the association between weekly frequency of participation and CVD- and cancer-related mortality for all activities combined, as well as for receptive and creative activities, respectively. Weekly participation in creative activities significantly reduced the risk of CVD-related mortality; those participating every other week or less than once per week and those participating once to less than twice per week had a 36% (HR: 0.64; 99% CI: 0.46–0.89) and 26% (HR: 0.74; 99% CI: 0.57–0.96), respectively, lower risk of CVD-related death. Participating more than twice a week in any of the creative activities was not associated with a significantly lower risk of CVD-related mortality. While gender-specific analyses indicated similar trends for both genders in terms of risk reduction, statistical significance was found only among men who participated in creative activities every other week or less than once per week (40%; HR: 0.60; 99% CI: 0.39–0.93).

**Table 4:** Association, both overall and gender-specific, between CVD- and cancer-related mortality, respectively, and weekly frequency of participation in receptive, creative, and combined activities (based on adjusted^†^ hazard ratios and 99% confidence intervals: N = 35,902). The HUNT Study (2006–08).

Frequency/wk.			0.5–< 1	1–< 2.	≥ 2*
**CVD**	**Combined**	All	0.92 (0.66–1.29)	0.71 (0.55–1.01)	0.72 (0.52–1.01)
		Men	1.03 (0.65–1.63)	0.78 (0.51–1.19)	0.76 (0.48–1.19)
		Women	0.79 (0.48–1.29)	0.68 (0.43–1.07)	0.67 (0.41–1.11)
	**Receptive**	All	0.82 (0.63–1.07)	0.92 (0.69–1.22)	0.85 (0.33–2.15)
		Men	0.77 (0.54–1.09)	0.84 (0.58–1.22)	0.69 (0.18–2.56)
		Women	0.88 (0.59–1.31)	1.05 (0.67–1.65)	1.43 (0.38–5.32)
	**Creative**	All	0.64 (0.46–0.89)	0.74 (0.57–0.96)	0.90 (0.56–1.44)
		Men	0.60 (0.39–0.93)	0.77 (0.55–1.08)	0.80 (0.43–1.51)
		Women	0.67 (0.41–1.09)	0.68 (0.45–1.03)	0.96 (0.47–1.96)
**Cancer**	**Combined**	All	0.90 (0.65–1.23)	0.78 (0.59–1.04)	0.71 (0.53–0.97)
		Men	0.94 (0.62–1.42)	0.77 (0.53–1.12)	0.70 (0.47–1.04)
		Women	0.85 (0.52–1.40)	0.80 (0.52–1.25)	0.74 (0.46–1.18)
	**Receptive**	All	0.81 (0.64–1.01)	0.86 (0.67–1.11)	1.15 (0.56–2.41)
		Men	0.83 (0.62–1.12)	0.87 (0.63–1.20)	1.18 (0.46–3.01)
		Women	0.79 (0.55–1.12)	0.85 (0.58–1.26)	1.07 (0.33–3.47)
	**Creative**	All	0.80 (0.62–1.05)	0.74 (0.59–0.93)	0.66 (0.43–1.02)
		Men	0.78 (0.55–1.11)	0.75 (0.56–1.02)	0.74 (0.43–1.27)
		Women	0.84 (0.56,1.27)	0.71 (0.49–1.02)	0.58 (0.29–1.17)

^†^ Respective frequency max: four times/wk; creative frequency max: five times/wk; combined frequency max: nine times/wk.

Fully adjusted ref.: never or seldom.

In contrast, for cancer-related mortality significant reductions, after full adjustment, were observed when weekly frequency of participation in combined activities was more than twice a week. In other words, when creative and receptive activities were combined, a significantly lower HR of cancer-related mortality was found (HR: 0.71; 99% CI: 0.53–0.97).

## Discussion

The results of this observational cohort study suggest that participation in cultural life is associated with a reduced risk of CVD-related death. In particular, our results indicate that frequent weekly participation in creative activities reduces the risk of CVD- and cancer-related mortality. When receptive and creative activities were combined, a significantly lower HR for cancer-related mortality was found for the sample (both genders included), but only if the frequency of participation was over twice a week; this is probably attributable to creative activity participation. Further, our results indicated that diversity of participation does not influence this association.

Before discussing our findings in the context of other studies, it is important to note that methodological differences between studies, such as the types of cultural activities examined, the operationalisation of the exposure measures (i.e. measuring individual activities or frequency or diversity of participation), and the outcomes measured, greatly impact inter-study comparisons. Another important issue is the difficulty separating the total number of activities from the frequency of participation, as it is likely that, the more activities a person performs, the higher his/her frequency score. These two quantifiers of the diversity and frequency of cultural-activity engagement are not mutually exclusive and have seldom been implemented in other studies. We observed that in previous published articles, the most common method was to combine the number of activities and frequency in the same index.

A previous Scandinavia-based prospective study examined similar outcomes to the present research, and used an index that combined frequency of participation in receptive activities (attending a cinema, theatre, art gallery, museum, and live music) and the number of these activities attended. The study found that those who live in urban areas and rarely attend cultural events have a threefold higher risk of cancer-related mortality when compared to frequent attendees [23]. In contrast, we did not find an association between reduced cancer-related mortality and frequent attendance of receptive activities, unless creative activities were also performed. Contrary to our finding, Fancourt et al. studied the association between all-cause mortality among adults aged 50 years and above and found that those who engaged with receptive arts activities even on an infrequent basis such as every few months or more had 31% lower mortality rate compared to those who never engaged in such activities. This finding was independent of demographic, SES, health related behaviour and social factor and after adjustment for their cognitive status, mental health and PA [32].

In our cohort, participation in outdoor activities and club meetings was strongly associated with CVD-related mortality, while parish work, singing or playing music, and dancing were not. Väänänen et al. [24] studied, among a cohort of Finnish industrial employees (n = 7,922), the association between engagement in cultural activities (arts and culture, association activities, societal actions, reading literature, and studying) and main causes of mortality. High engagement (i.e. approximately twice a month to daily) was associated with a 32% lower risk of CVD-related mortality. There are several differences between these findings and those of the present study. The creative activities examined by Väänänen et al. differed from those measured in HUNT3; we found a reduced risk of CVD-related mortality among those who participated in creative activities as infrequently as less than once a week; and Väänänen et al. did not find any associations with cancer-related mortality [24].

Also contrasting with our results, Merom et al. [22] found dancing to be inversely associated with CVD-related mortality. Specifically, they found moderate-intensity dancing to be associated with a lower risk of CVD-related mortality to an greater extent than moderate-intensity walking (46% versus 33%, respectively). Outdoor activity is positively related to PA, which is an established protective factor against mortality from CVD and from some types of cancer. Donneyong et al. [33] found that outdoor activity is strongly associated with a reduced risk of CVD-related mortality (30–47%, depending on participation frequency), and later reported a risk reduction of 28% independent of total PA [34]. These results are stronger than those obtained for our cohort, which showed that outdoor-activity participation produces a risk reduction of 23%. Outdoor activities may be a marker of an active lifestyle, and reduced sedentary behaviour. However, engagement in such activities does not necessarily involve PA, and outdoor activity is significantly different from exercise. Engagement in outdoor activity influences levels of inactivity and sedentarism, both of which are contributors to chronic diseases [35, 36] The joint association between PA and sedentary behaviour has been intensively explored in the past decade [37]. Rangul et al. [38] found no evidence that people who spend prolonged periods seated or who have low levels of PA have an increased risk of total cancer incidence when compared to people who spend short periods seated or who are physically active. Autenrieth et al. [39] found that PA during leisure time is associated with cancer-related mortality, and vigorous activity with CVD-related mortality. In contrast, a systematic review found significant associations between sedentary behaviour and cancer [40]. Sedentary behaviour and physical inactivity are associated with a risk of several chronic diseases [41–43], and are of importance in regard to incident CVD [44] and cancer, especially colon and breast cancer [41]. Stamatakis, E et.al. revealed that by replacing one hour sedentary time with walking, led to a 14% reduced risk of all-cause mortality [45]. Another explanation for the strong association between outdoor activity and reduced CVD risk is that outdoor activity involves exposure to natural environments and provides opportunities for positive restoration [46]. The level of energy expenditure associated with outdoor activities can vary; in the present study, we adjusted for energy expenditure, but an attributable effect on CVD risk remained. Another possible explanation is that outdoor activities increase sunlight exposure, which can prevent autoimmune diseases, CVD, and cancers [47]. Sunlight exposure counteracts vitamin-D deficiency, which is associated with increased risk of CVD [47], deadly cancers [47, 48], and nonmelanoma skin cancer [47]. However, Donneyoung et al. found CVD-related mortality risk to be independent of vitamin-D level [33, 34].

Among receptive activities, only attending museum and art exhibitions appeared to protect against CVD -related mortality, but none protected against cancer-related mortality. To our knowledge, museum visits has not been previously explored as an activity related to mortality; but instead, it has been considered in relation to general health and wellbeing [49–52] or cognitive decline and the prevention of dementia. A longitudinal study by Fancourt et al. [49] found that visiting a museum every few months is related to lower incidence of dementia in adults aged > 50 years. A possible explanation is that visiting museums reduces the negative effect of possible sedentary behaviours and isolation, and can also represent social engagement [49]. In general, a lack of social support is known to cause negative psychological states, such as anxiety and depression, which further can increase the risk of CVD [53]. A previous study performed a tactile experiment in which participants handled and discussed a selection of museum objects and discussed photographs of the same objects; this activity enhanced cancer patients’ well-being, positive emotions, and happiness [54]. However, we found no association between attending museums or art exhibitions and cancer-related mortality.

Music, singing, and theatre engagement was the only creative activity that was significantly associated with a reduced risk of cancer-related mortality. This findings is supported by many clinical studies that have shown such activity to have a therapeutic effect on cancer patients. Music has been linked to immune response [13, 55, 56], with stress reduction as a possible pathway, and may impact individuals’ neurological and immunological systems [56]. However, there is a lack of epidemiological studies in this regard. Our results, therefore, the first to show that music, singing, and theatre participation reduces the risk of cancer-related mortality by 27% in the general population; this should have implications for future research.

There is a strong empirical rationale for our exploring of gender differences. First, patterns and durations of diseases can differ across genders [57]. Second, there is evidence that behavioural choices and the time allocated to making these choices differ by gender [58]. In our analysis, gender was a significant covariate for each exposure variable (i.e. never/ever, number of activities, and frequency). Specifying interaction terms between cultural participation and sex, we found statistically significant differences for ‘association and club meeting activities’ and parish work for CVD-related mortality, and ‘museum and art exhibition’ and dance for cancer-related mortality. We found that club meetings reduce the risk of CVD-related mortality among women by 36%, and that music, singing, and theatre engagement may reduce cancer-related mortality among men by 27%. Further, men who engaged in creative activities less than once a week showed a significantly (33%) lower risk of CVD-related mortality, whereas, for women the weekly frequency did not seem to be of importance. We are not aware of any other studies that have examined gender differences in relation to frequency of cultural participation and CVD-related mortality. A women-only cohort found that frequent attendance of religious services is associated with a significantly lower risk of CVD- and cancer-related mortality, when compared to never attending religious services; women who attend more than once a week have a 27% and 21% lower risk of CVD-related mortality and cancer-related mortality, respectively [59]. In our study, parish work was associated with a reduced risk of CVD-related mortality by 47% among women, while this was not statistically significant which could be due to small sample reporting parish work, (n = 1410) it is a strong protective effect size. Similar to our findings, Eng et al. did not find religious-service attendance to be significantly associated with reduced CVD-related mortality among men [6].

Causal pathways in health are presumably complex, and there are several risk factors for disease onset and mortality. Stress is strongly associated with CVD incidence [60], and is associated with depression and metabolic abnormalities that increase CVD risk [61]; further, chronic psychosocial stress modifies the association between inflammation and CVD [62]. A previous meta-analysis found chronic stressors to be associated with suppression of cellular and hormonal measures [63]. Additionally, stress may promote the initiation and progression of some types of cancer [64], thereby influencing cancer-related mortality [23]. The immune system and stress response seem to be of particular importance in regard to cancer [64–66], with immunological involvement varying across different cancers [64]. Psychological stress, both among healthy individuals experiencing stress and individuals with cancer-related psychological stress, is linked to the downregulation of immune responses immune responses, which has implications regarding cancer progression. Engaging in cultural events could promote immune functions by serving as a buffer against stress [23]. For example, interventions involving dance-movement therapy groups have shown positive effects regarding stress reduction [67], and art therapy has been found to increase overall coping resources among women with breast cancer [18]. A physically active lifestyle strengthens the ability to manage stress exposure and stress-related disorders [68]; in particular, cardiorespiratory fitness moderates stress and seems to be associated with fewer symptoms of depression and burnout [68]. Cultural activities can counteract adverse stress-related effects by promoting social networks and resilience [69, 70]. We suggest new studies regarding fair access to participation in cultural life, not least in view of the possible stress reducing effects from cultural engagement.

In observational studies such as this, causality is difficult to demonstrate. Cultural participation may serve as a proxy for other factors [71], such as social capital and factors related to SES. Fancourt and Steptoe explored cultural engagement in relation to mental health, and found it to be independent of socioeconomic status [72]. Another important public health aspect is the mental health challenges in the population. Anxiety and depression are prevalent conditions and found to be barriers for cultural engagement by Fancourt et al. [73]. Those participating in cultural activities may also be a healthier population than non-participants, and frequent attendees may be healthier than those who seldom participate. When compared to less-active people, such people may have greater knowledge, network support (representing a support mechanism), and ability to take advantage of knowledge regarding lifestyle and treatment options, and some may have access to private health-care financing through private insurance. Disease onset may encourage people to adopt healthier lifestyles, strengthening factors that improve their psychological and physical health, well-being, and quality of life, helping them to enjoy life.

### Strengths and limitations

This study involved data for a large population-representative cohort that had an acceptable response rate and obtained rich information regarding a range of receptive and creative activities The survey collected information on important confounders. A major strength is this research’s exploration of the risk of cause-specific mortality fora range of receptive and creative activities. Participants were blinded to future research questions when invited to participate in the HUNT3 Survey, which reduced social-desirability bias. Lastly, the use of cause of death data from the national register provided high degrees of coverage and completeness regarding cause-specific mortality.

Limitations include a lack of adjustment for changes over the follow-up period in relevant characteristics, health, and behaviours; further, possible joint effects of multiple risk factors (e.g. a cluster of risk factors within a single individual) and comorbidities were not examined. Some of the activities could not be separated (e.g. music, singing, and theatre). In addition, isolating activities is complex, as participating in creative activities could increase the likelihood of attending a concert, theatre, and/or cinema, or vice-versa. Consequently, the single-effect estimates may be confounded and may have measured attributable effects from other activities. Furthermore, other activities may be more strongly linked to sub-causes of mortality within the CVD and cancer categories. Statistically, we took a conservative approach by presenting 99% CIs; this was because we performed multiple testing and used 99% CIs to limit the type I error rate.

## Conclusion

The results of this study have important implications for research, leisure-service providers and policy-makers. Researchers should continue to explore casual paths between stress reducing, social capital and mental well-being effects from cultural engagement among the general population. Leisure-service providers should increase the opportunities to engage in outdoor recreational activities, increase the number of clubs with affordable memberships, and create more opportunities to consistently engage in music, singing, and theatre. Policy-makers should review whether there is sufficient access to museums and artistic events across all regions of the country. Such activities will increase social interaction in the community, foster psychosocial benefits and, hopefully, promote and maintain health and enhance longevity.

## Supporting information

S1 Fig Diversity of receptive activities in association with CVD mortality, n = 35,902. The HUNT Study (2006–08). *Number of activities from 1, 2 or maximum 3–4 activities attended, for the total sample and stratified for genders.
(TIF)

S2 Fig Diversity of creative activities in association with CVD mortality, n = 35,902. The HUNT Study (2006–08). *Number of activities from 1, 2 or maximum 3–5 activities engaged in, for the total sample and stratified for genders.
(TIF)

S3 Fig Total diversity of activities, combined receptive and creative activities, in association with CVD mortality, n = 35,902. *Number of activities from 1, 2 and up to maximum 7–9 activities engaged in, for the total sample and stratified for genders.
(TIF)

S4 Fig Diversity of receptive activities in association with cancer mortality, n = 35,902. The HUNT Study (2006–08). *Number of activities from 1, 2 or maximum 3–4 activities engaged in, for the total sample and stratified for genders.
(TIF)

S5 Fig Diversity of creative activities in association with cancer mortality, n = 35,902. The HUNT Study (2006–08). *Number of activities from 1, 2 or maximum 3–5 activities engaged in, for the total sample and stratified for genders.
(TIF)

S6 Fig Total diversity of activities, combined receptive and creative activities, in association with cancer mortality, n = 35,902. The HUNT Study (2006–08). *Number of activities from 1, 2 up to maximum 7–9 activities engaged in, for the total sample and stratified for genders.
(TIF)
