# Supplementary figures and images for: Association of engagement in cultural activities with cause-specific mortality determined through an eight-year follow up: The HUNT Study, Norway
Source: PLoS One. 2021 Mar 11;16(3):e0248332. doi: 10.1371/journal.pone.0248332 (PMC7951895; doi:10.1371/journal.pone.0248332)

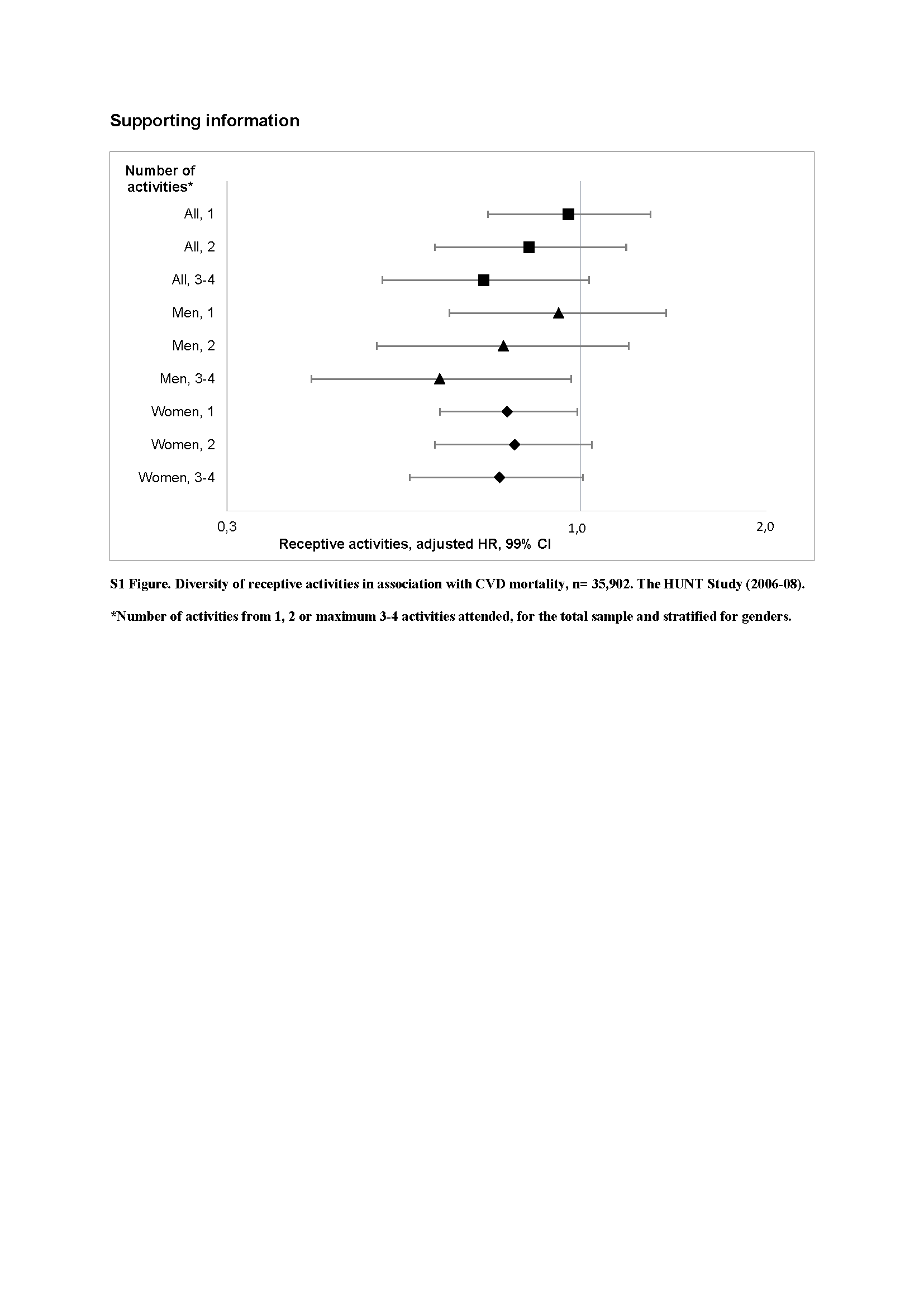

Supplement: S1 Fig — The HUNT Study (2006–08). *Number of activities from 1, 2 or maximum 3–4 activities attended, for the total sample and stratified for genders. (TIF) [file pone.0248332.s001.tif]

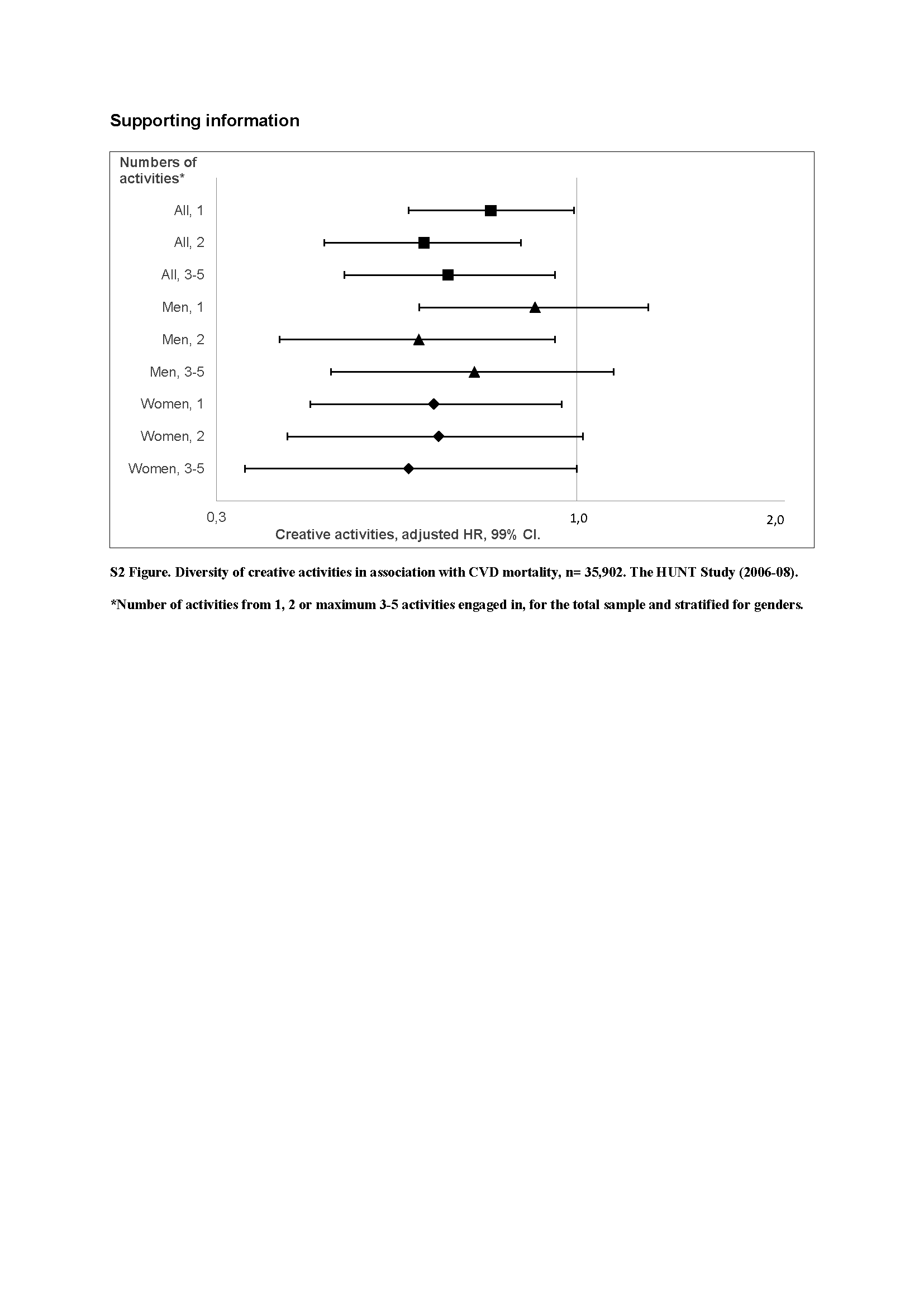

Supplement: S2 Fig — The HUNT Study (2006–08). *Number of activities from 1, 2 or maximum 3–5 activities engaged in, for the total sample and stratified for genders. (TIF) [file pone.0248332.s002.tif]

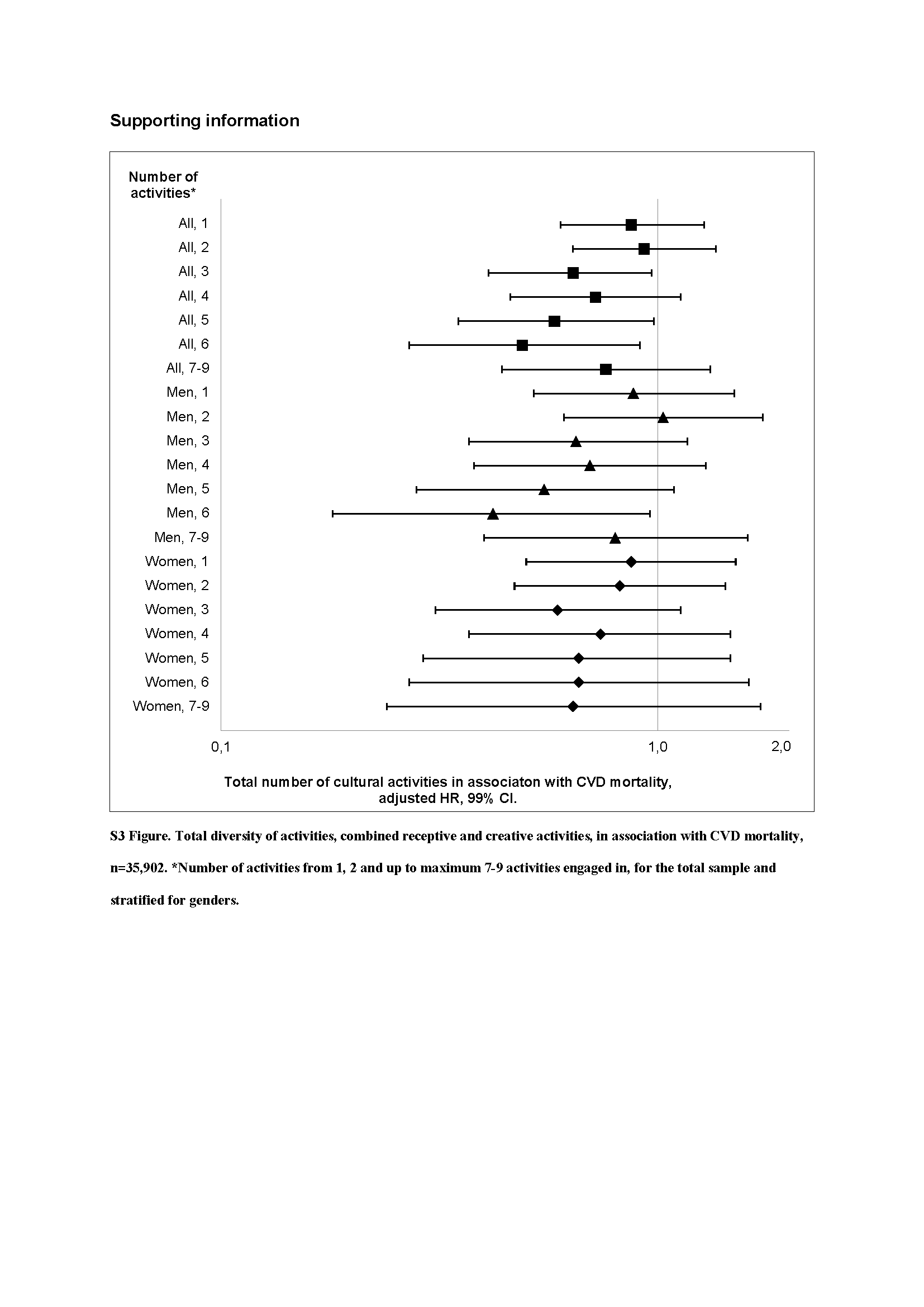

Supplement: S3 Fig — *Number of activities from 1, 2 and up to maximum 7–9 activities engaged in, for the total sample and stratified for genders. (TIF) [file pone.0248332.s003.tif]

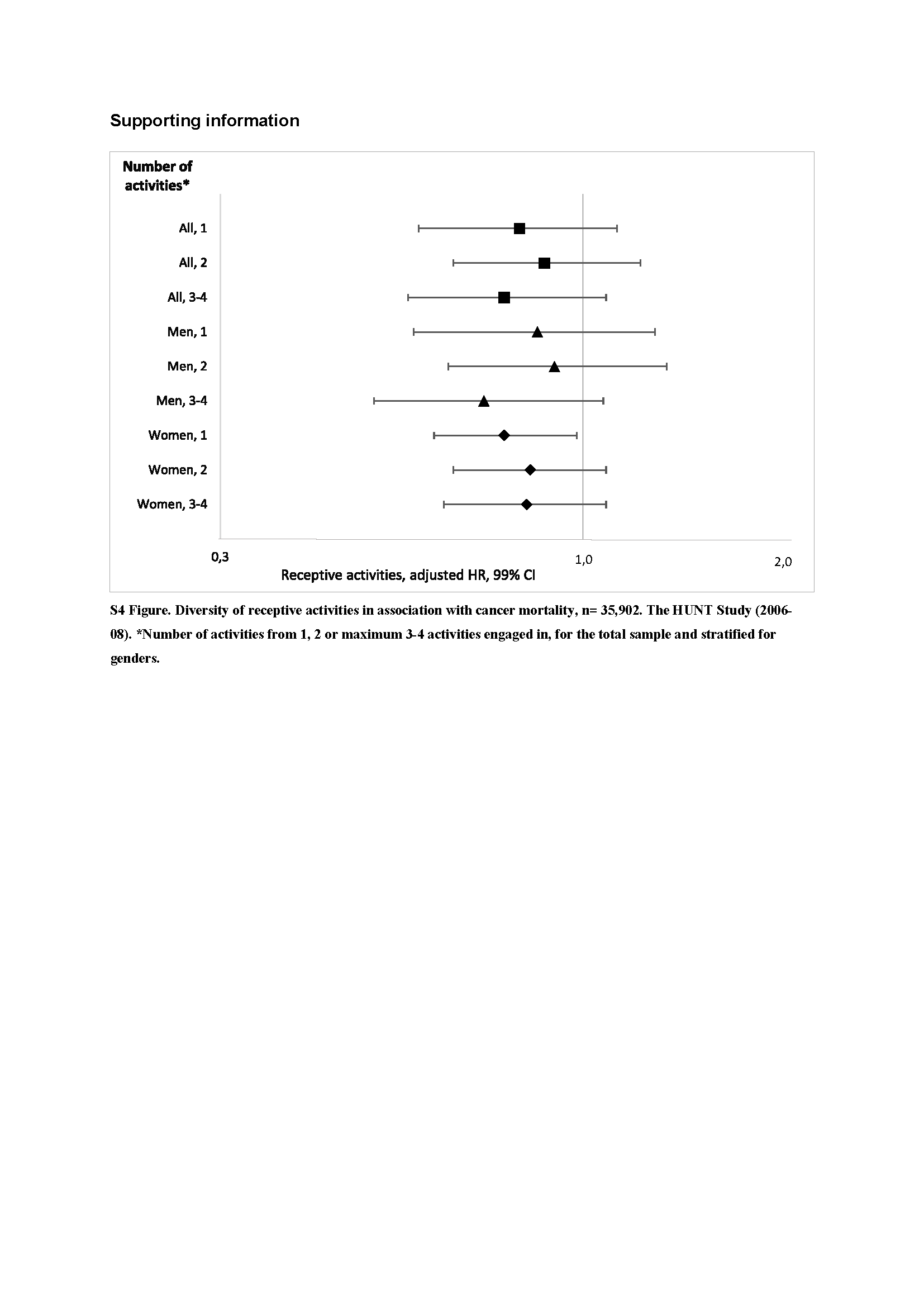

Supplement: S4 Fig — The HUNT Study (2006–08). *Number of activities from 1, 2 or maximum 3–4 activities engaged in, for the total sample and stratified for genders. (TIF) [file pone.0248332.s004.tif]

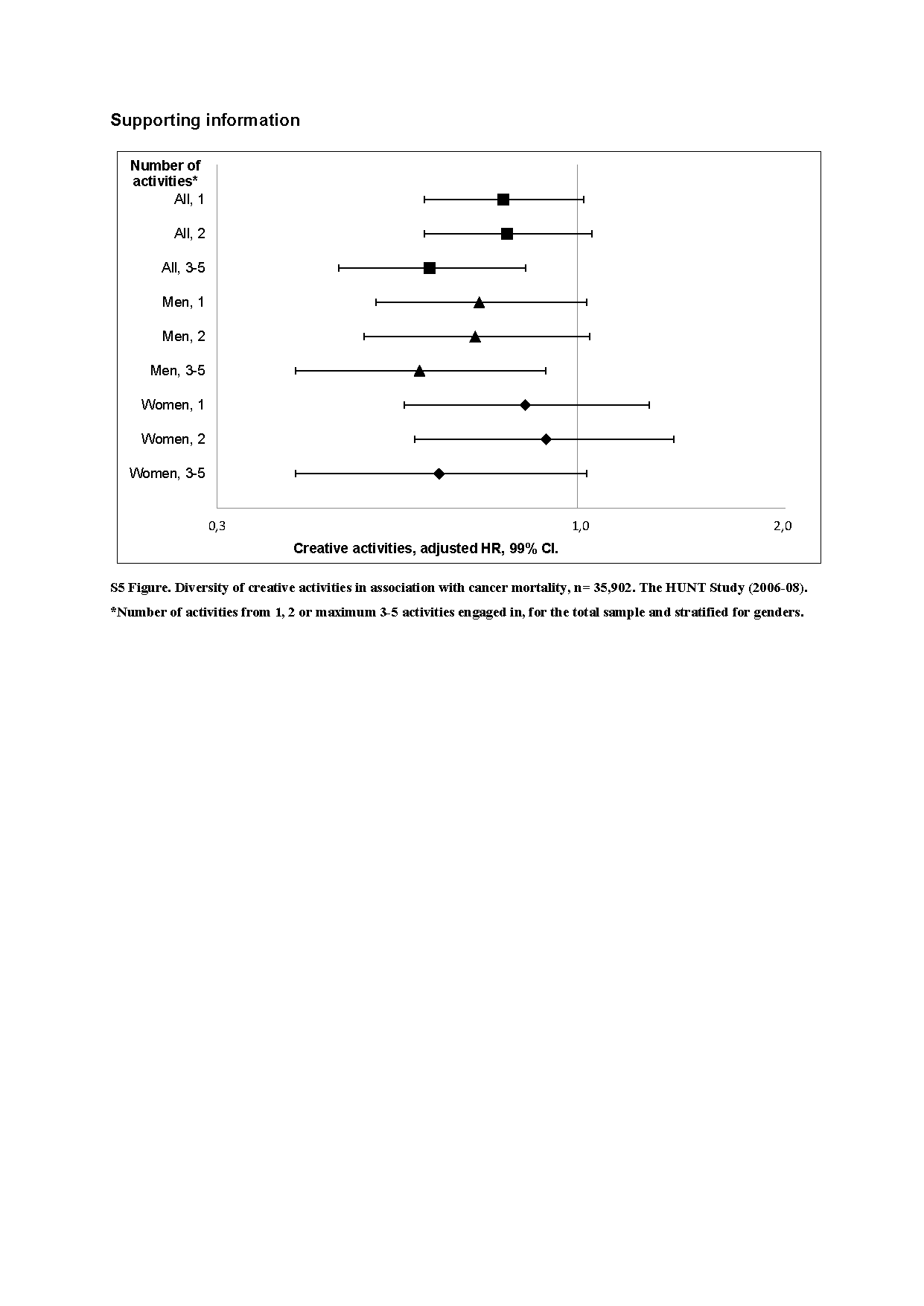

Supplement: S5 Fig — The HUNT Study (2006–08). *Number of activities from 1, 2 or maximum 3–5 activities engaged in, for the total sample and stratified for genders. (TIF) [file pone.0248332.s005.tif]

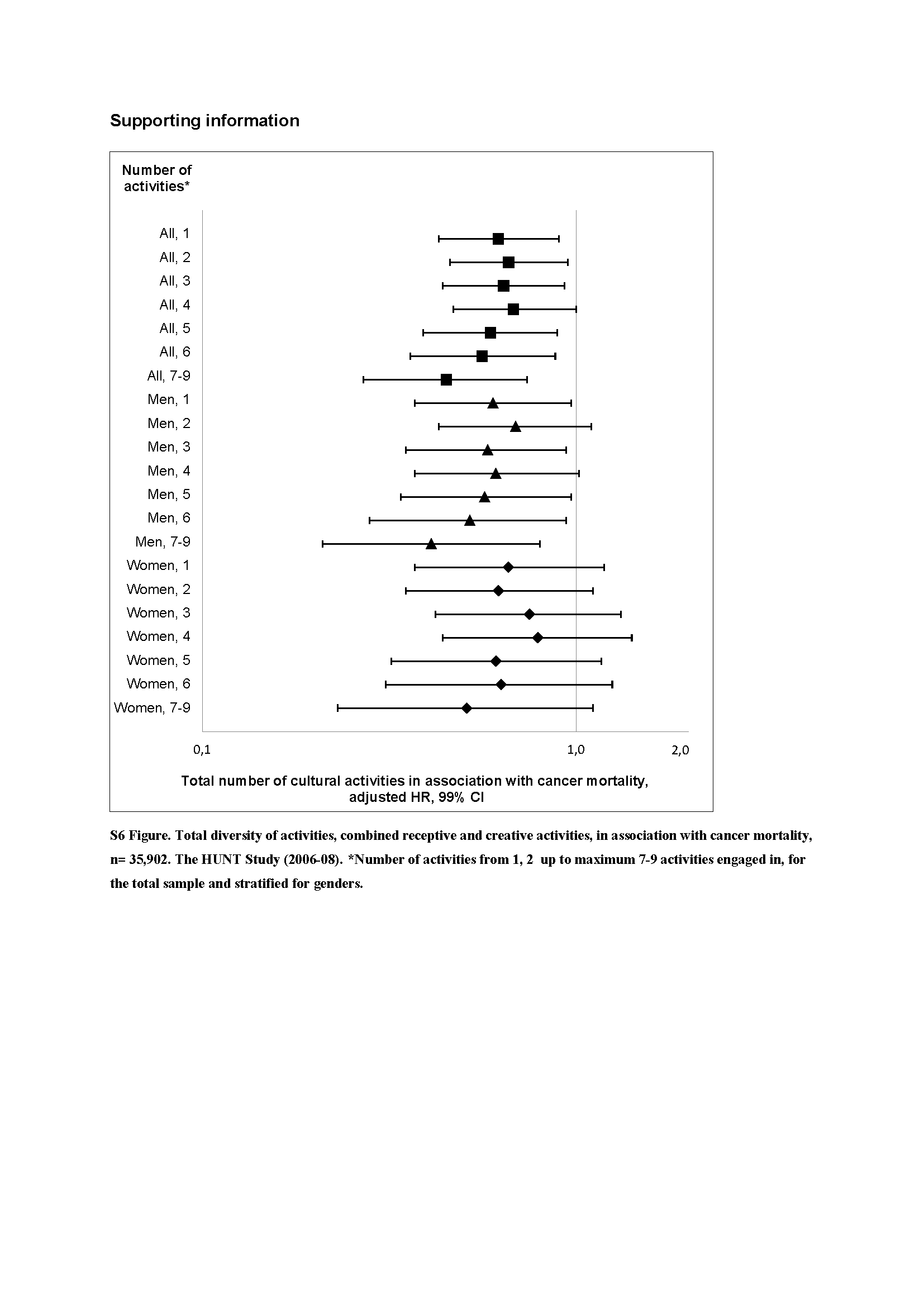

Supplement: S6 Fig — The HUNT Study (2006–08). *Number of activities from 1, 2 up to maximum 7–9 activities engaged in, for the total sample and stratified for genders. (TIF) [file pone.0248332.s006.tif]
